# Modifying and Integrating *in vitro* and *ex vivo* Respiratory Models for Inhalation Drug Screening

**DOI:** 10.3389/fbioe.2020.581995

**Published:** 2020-10-23

**Authors:** Aylin Cidem, Peta Bradbury, Daniela Traini, Hui Xin Ong

**Affiliations:** ^1^Respiratory Technology, Woolcock Institute of Medical Research, Sydney, NSW, Australia; ^2^Faculty of Medicine and Health, University of Sydney, Sydney, NSW, Australia

**Keywords:** drug efficacy, drug delivery, drug toxicity, inhalation therapy, isolated perfused lung, lung-on-chip, organoid, precision-cut lung slices

## Abstract

For the past 50 years, the route of inhalation has been utilized to administer therapies to treat a variety of respiratory and pulmonary diseases. When compared with other drug administration routes, inhalation offers a targeted, non-invasive approach to deliver rapid onset of drug action to the lung, minimizing systemic drug exposure and subsequent side effects. However, despite advances in inhaled therapies, there is still a need to improve the preclinical screening and the efficacy of inhaled therapeutics. Innovative *in vitro* models of respiratory physiology to determine therapeutic efficacy of inhaled compounds have included the use of organoids, micro-engineered lung-on-chip systems and sophisticated bench-top platforms to enable a better understanding of pulmonary mechanisms at the molecular level, rapidly progressing inhaled therapeutic candidates to the clinic. Furthermore, the integration of complementary *ex vivo* models, such as precision-cut lung slices (PCLS) and isolated perfused lung platforms have further advanced preclinical drug screening approaches by providing *in vivo* relevance. In this review, we address the challenges and advances of *in vitro* models and discuss the implementation of *ex vivo* inhaled drug screening models. Specifically, we address the importance of understanding human *in vivo* pulmonary mechanisms in assessing strategies of the preclinical screening of drug efficacy, toxicity and delivery of inhaled therapeutics.

## Introduction

Respiratory diseases are among the leading causes of mortality worldwide, with chronic obstructive pulmonary disease (COPD), lung infections (viral and bacterial), lung cancer and tuberculosis all listed in the top 10 causes of death (World Health Organisation [WHO], 2019a, b). Respiratory diseases impose an immense global health burden, with an estimated 1 billion people suffering from either acute or chronic conditions that result in upwards of 4 million deaths annually (World Health Organisation [WHO], 2014). Treatment of respiratory diseases relies on a variety of drug administration routes, however, not all routes are effective for disease or symptomatic relief. For example, when treating lung cancer, anti-cancer therapeutics are commonly administered systemically, resulting in low drug concentrations at the tumor site, reduced efficacy and multiple negative systemic side effects (Palumbo et al., 2013). Thus, a more targeted approach to the delivery and administration of anti-cancer drugs for lung cancer patients may increase therapeutic benefit and also quality of life. Whereas, oral and intravenous drug administration of high dose antibiotics and anti-inflammatory agents are commonly used to treat COPD, cystic fibrosis, pulmonary oedemas, and respiratory infections. However, both of these administration routes require the drug to be pre-processed by either liver or kidney and as a result, sustained long-term treatment strategies often cause liver/kidney toxicity and failure (Ditchfield et al., 2018). As such, a targeted delivery approach that deposits drugs directly within the lung (via inhalation) will distribute optimal and effective drug concentrations to the diseased site, minimize systemic drug exposure by negating any negative side effects, and thereby improve therapeutic efficacy, patient outcomes and patient quality of life (Borghardt et al., 2018).

The complex anatomical structure and branching of the lung present a unique challenge when attempting a targeted drug delivery approach. The airways also contain several biological barriers and properties that can limit drug uptake (transport, absorption) and subsequent efficacy (Ruge et al., 2013). Following inhalation, drugs deposited in the lung are subject to removal by the natural clearance mechanisms of the epithelium; goblet cells secrete a mucous layer to line the airways, and ciliated cells rapidly beat in a coordinated, metachronous fashion to move the mucous-trapped drug particle up, and out of the airway (Eliezer et al., 1970). While, drugs deposited in lower lung regions, for example in the alveolar space, are engulfed and removed by resident alveolar macrophages (Wanner et al., 1996; Ruge et al., 2013). However, once a drug is successfully deposited within an airway, a new challenge arises, as the epithelial cells that line the bronchial tree form tight junctions and it is these tight junctions that limit drug uptake and therefore therapeutic efficacy (Ghadiri et al., 2019). Importantly, however, it is not just the structural, physiological and biological properties of the lung that present challenges for inhaled therapies, the physiochemical and pharmacokinetic characteristics of the drug also need to be taken into account.

The physiochemical attributes of an inhaled drug interact with the biological properties of a lung to determine the deposition site, mechanism of action and therapeutic efficacy. The size of an aerosolized drug particle plays a pivotal role in determining the exact deposition site within the lung with particles of an aerodynamic diameter of 5–10 μm depositing in the larger airways, while particles less than 2 μm in diameter can be targeted to the bronchoalveolar and deep lung alveolar regions (Davies and Muir, 1966; Yeh et al., 1976; Lippmann et al., 1980). Particles with an aerodynamic diameter smaller than 0.5 μm can theoretically be delivered to the alveolar space, however, the majority of particles are immediately exhaled following inhalation due to their small size (Davies and Muir, 1966; Yeh et al., 1976; Lippmann et al., 1980). In order to evade the aforementioned clearance mechanisms of the airways, inhaled therapeutics are engineered with the appropriate surface chemistry to avoid adhesion to the mucosal layer and yet small enough for deposition beyond the large airways (Lippmann et al., 1980).

To appropriately determine key parameters of inhaled therapies (aerodynamic performance, pharmacodynamics and pharmacokinetics), *in vitro* lung models have been developed, validated and characterized to be used as preclinical screening tools. One of the key objectives for these *in vitro* research models is to mimic the structural and biological properties of the *in vivo* human lung environment to better replicate therapeutic exposure, deposition and efficacies. It is important to note that the translation of results from human *in vitro* models to *in vivo* animal (namely, rodent and pig) experimental results have proved limited and is hypothesized to be due to the anatomical, physiological and pathophysiological differences of human and animal lungs (Ware, 2008; Aun et al., 2017). In this review, we address the challenges and advances in *in vitro* and *ex vivo* respiratory research models when evaluating therapeutic efficacy of inhaled therapeutics in a preclinical setting to ensure successful translation to the clinic. Specifically, we discuss the implementation of progressive lung models (respiratory organoids, lung-on-chip platforms) and sophisticated bench-top approaches to evaluate inhaled drug delivery, efficacy and potential cytotoxicity.

## Conventional Respiratory *in vitro* Cell Models

### Mimicking the Heterogeneity of the Respiratory Microenvironment

The respiratory epithelium lines the airways and provides the principal physical barrier to transport and absorption of foreign particles, including inhaled therapies. The bronchial epithelium is composed of a heterogeneous mix of cells that stem from distinctive lung progenitor cells and can self-renew and differentiate into goblet, ciliated, or basal cells (Eliezer et al., 1970; Wanner et al., 1996; Ruge et al., 2013; Li et al., 2015). As mentioned previously, these differentiated epithelia cells protect the respiratory tract from foreign irritants such as smoke and dust, but also drug particles (Wanner et al., 1996). Epithelial cells form tight junctions between the neighboring epithelial cells to seal off the paracellular space between cells, regulating the influx and efflux of xenobiotics, but also the release of inflammatory mediators following inhalation of foreign agents, irritants and particulates to activate and recruit immune cells (Madara, 1998). Thus, mimicking these important biological barriers and properties *in vitro* has become an important tool when validating the appropriateness of a cell-based model. Numerous human bronchial epithelial cell lines have been used to recapitulate both healthy and diseased respiratory environments *in vitro to specifically evaluate drug transport in response to the biological barriers of the epithelium* (16HBE16o-, NuLi-1, and BEAS-2B (healthy human bronchial epithelial cells; Forbes et al., 2003; Garcia-Canton et al., 2013; Monnappa et al., 2016), Calu-3 and NCI-H441 (lung adenocarcinoma; Ong et al., 2011; Salomon et al., 2014); and CuFi-1 and CuFi-5 (cystic fibrosis; Molina et al., 2015; Sheikh et al., 2020).

### Progress and Limitations of *in vitro* Air-Liquid Interface Models

To recapitulate luminal airflow of the respiratory system *in vitro*, an air-liquid interface (ALI) culture system has been implemented. Briefly, cells are first seeded onto the semi-permeable membrane of a Transwell support and both the apical (upper; cell) and basolateral (bottom; media) chambers are submerged in culture medium. When cell confluency is reached, media from the apical chamber removed, exposing the cultured cells to air and therefore establishing the ALI. Importantly, exposing cells to the air forces the cells to differentiate, secrete mucus and establish tight junctions, mimicking the *in vivo* respiratory environment. Furthermore, ALI respiratory models allow *in vivo* inhaled drug exposure conditions to be replicated by subjecting drug particles to the differentiated cell layer of the model. As such, ALI culture models provide a unique *in vitro* platform to mimic drug deposition onto the respiratory epithelial surface allowing downstream drug transport, efficacy and cytotoxicity studies to be performed (Grainger et al., 2006; Ong et al., 2011; Salomon et al., 2014).

Formation of the key biological parameters, namely tight junctions and differentiation is required for a successful ALI culture model to appropriately assess drug uptake, solute permeability and transport mechanisms. Various immortalized respiratory cell lines, including Calu-3, A549, 16HBE, NuLi-1, CuFi-1, and NCI-H441, have all been shown to form tight junctions and/or produce mucous under specific and appropriate ALI culture conditions (Grainger et al., 2006; Ong et al., 2011; Salomon et al., 2014; Faura Tellez et al., 2016; Latvala et al., 2016; Sheikh et al., 2020). However, many cell lines are limited in their ability to functionally recapitulate the *in vivo* respiratory epithelium. For example, the A549 cell line is unable to form functional tight junctions as A549 cells show reduced expression of the seal-forming proteins, claudin-3, -4, and -5 (Ren et al., 2016), while the Calu-3 cell line is unable to produce functional ciliary activity (Kreft et al., 2015). As a result, ALI culture systems have progressed to use primary human respiratory epithelial cells as primary cells can generate ciliated cells, mucus secretions and form tight junctions to better represent the *in vivo* respiratory epithelium (Pezzulo et al., 2011; Wang et al., 2018). However, there are several caveats to the use of primary epithelial cells as these cells have a finite population doubling and obtaining primary human respiratory epithelial cells from commercial sources is often expensive. As an alternative, primary bronchial epithelial cells can be harvested and expanded from deceased or transplanted human lungs, but this requires ethics approval and is a labor- and skill-intensive protocol. Despite these limitations, primary respiratory epithelial ALI culture models have been used to recapitulate *in vivo* airway epithelia as a biologically relevant *in vitro* drug screening platform (Ong et al., 2016). ALI culture systems have been heavily reviewed in the literature and have delivered highly impactful research outcomes (Movia et al., 2018; Upadhyay and Palmberg, 2018) however, the use of *in vitro* ALI models to determine toxicity, delivery and efficacy of inhaled therapeutics requires greater physiological and anatomical relevance. Specifically, *in vitro* respiratory models that implement breathing mechanics, aerosol deposition, and the co-culture of different cell types allow greater mechanistic and efficacious insights of novel inhalable therapeutic compounds. Thus, *in vitro* models that consider the biological and physiological diversity of the respiratory system provide an appropriate preclinical screening tool to determine how the respiratory microenvironment dictates the therapeutic response of inhaled agents (Table 1).

**TABLE 1:** Overview of the advantages and disadvantages of *in vitro* models used for respiratory inhalation drug screening.

*In vitro* model	Applications	Respiratory disease model	Advantages	Disadvantages	References
Air-liquid interface	Drug efficacy, toxicity and transport/delivery studies	COPD, cystic fibrosis, lung cancer	Mimics respiratory tract of the lung when exposed to toxic and therapeutic agents. Dose of inhalation agents highly controllable. Can be implemented in drug transport/delivery studies using impactor technologies.	Restricted to a select range of respiratory cell lines to functionally recapitulate the *in vivo* airway epithelium. Use of primary cells may possess ethical and cell-culturing limitations.	Grainger et al., 2006, 2012; Ong et al., 2011, 2014; Haghi et al., 2012; Kumar et al., 2020
OrganoidS	Drug efficacy and toxicity studies	Lung cancer, fibrosis, cystic fibrosis, viral and bacterial infections	Capacity to mimic cellular heterogeneity of lung microenvironment. Recapitulates structural architecture and cellular interactions of lung microenvironment. Ability to be implemented in personalized medicine studies through the use of patient-derived organoids	Do not possess breathing mechanics essential in airflow. Do not possess anatomical structures essential for addressing mouth-to-airway transit of inhaled therapies.	Tan et al., 2017; Jung et al., 2019; Miller et al., 2019
Lung-on-chip	Drug toxicity and efficacy studies	Severe asthma, COPD, lung cancer	Able to reproduce key morphological and biological processes of lung airway barriers through emulating cellular stretching of the alveolar microenvironment. Ability to recapitulate mechanical and shear stresses that result from cyclical breathing. Possess cellular heterogeneity and vascular flow rates to simulate the lung *in vivo* microenvironment.	Inability to evaluate aerosolized bio-pharmacokinetics observed during the mouth-to-airway transit of inhaled particles.	Huh et al., 2010, 2012; Benam et al., 2016; Konar et al., 2016

## *In vitro* Experimental Models That Physiologically Mimic the Respiratory System to Screen Inhaled Therapies

**FIGURE 1:**
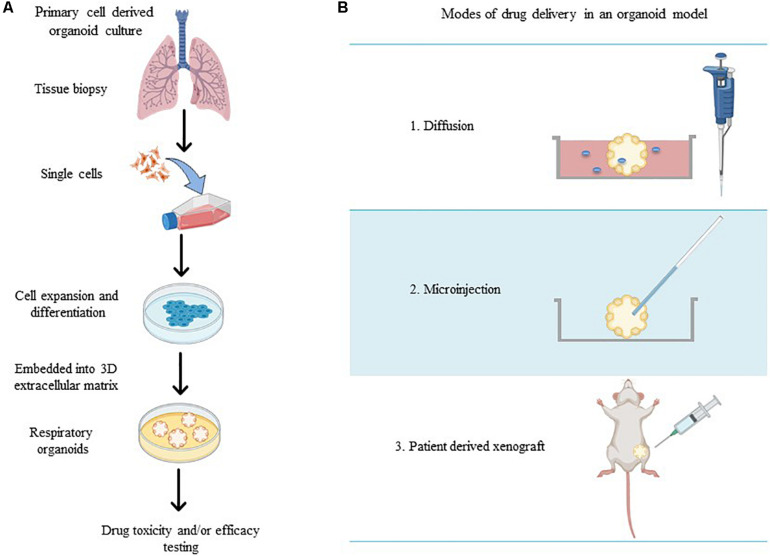
**(A)** Schematic overview of methods for the generation of respiratory organoid cultures derived from primary lung cells. **(B)** The varying modes of drug treatment delivery in an organoid model (made in ©BioRender - biorender.com).

**FIGURE 2:**
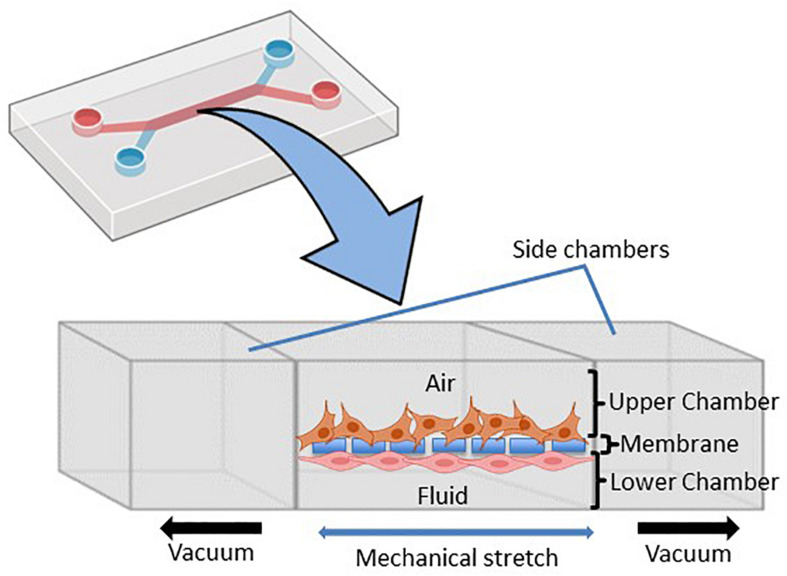
Schematic representation of the microfluidic lung-on-chip (LOC) system. Cross-section through the LOC model displaying the upper chamber consisting of human lung epithelial cells and the lower chamber consisting of pulmonary endothelial cells divided by a thin porous membrane. Side vacuum channels stretch out the membrane and mimic *in vivo* breathing-like forces (adapted from Huh et al., 2010 and made in ©BioRender - biorender.com).

**FIGURE 3:**
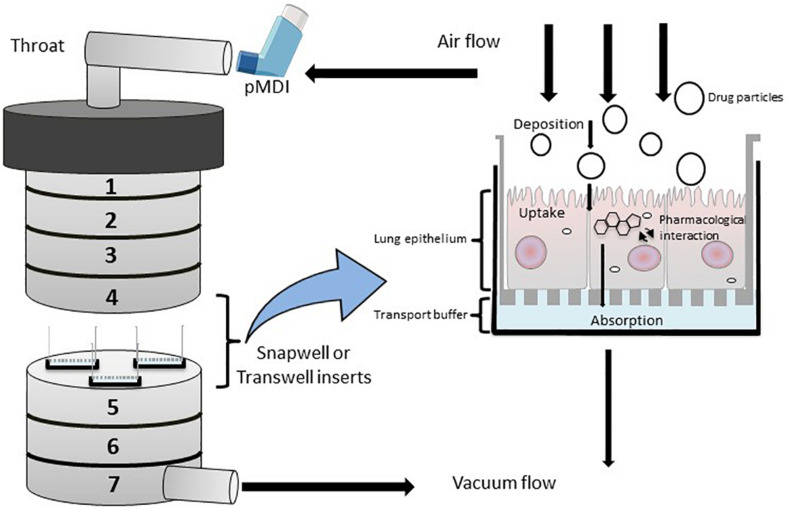
Diagrammatic rrepresentation of a modified Anderson Cascade Impactor (mACI) with the incorporation of Snapwell or Transwell inserts embedded with respiratory cell lines at the air-liquid interface (ALI). Airflow is maintained at a controlled flow rate and generated via vacuum flow on the opposite end of the mACI to simulate airflow and allow for the assessment of mouth-to-airway transit of drug particles. Evaluation of aerosolized drug particle deposition, transport and absorption across the cell epithelia is determined through assessing inserted Transwells.

**FIGURE 4:**
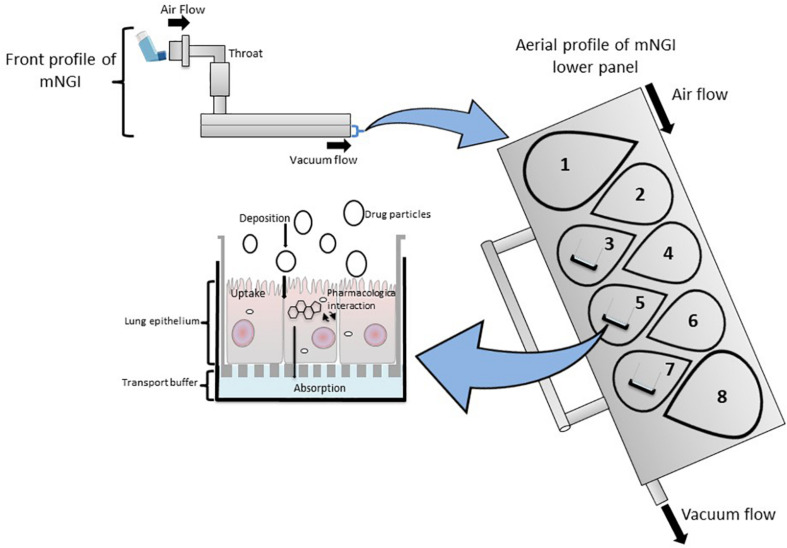
A modified Next Generation Impactor (mNGI) with the implementation of Transwell inserts embedded with respiratory cells on stages 3, 5, and 7 at the lower panel of the apparatus. Airflow is maintained at a controlled flow rate and generated via vacuum flow on the opposite end of the mNGI allowing for the assessment of mouth-to-airway transit of drug particles. Evaluation of aerosolized drug particle deposition, transport and absorption across the cell epithelia is determined through assessing inserted Transwells.

**FIGURE 5:**
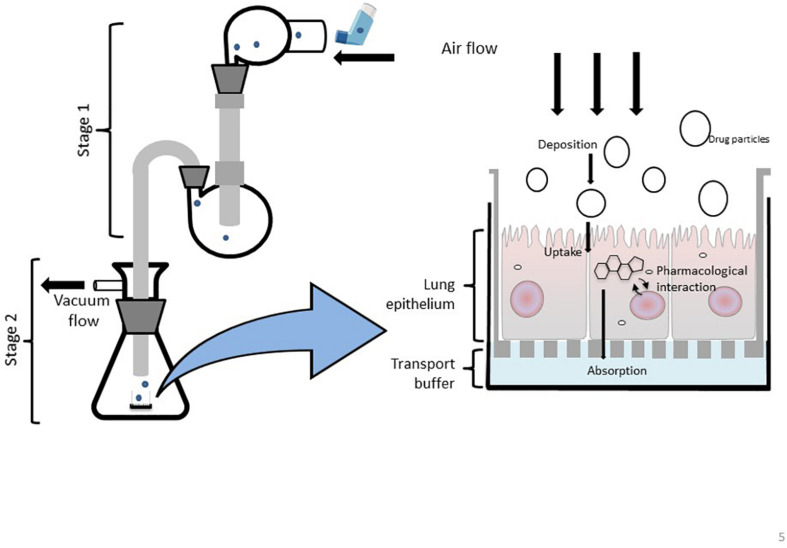
Schematic representation of a modified Twin Stage Impinger (mTSI) with the integration of ALI cell culture insert at the base of stage 2 enabling aerosolized drugs to be deposited directly at the respiratory epithelia to better mimic *in vivo* biopharmaceutical processes of particle deposition and absorption.

To accurately investigate drug delivery, efficacy, and toxicity of inhaled therapies, *in vitro* experimental models that accurately resemble the *in vivo* physiology are crucial research tools. As such, *in vitro* experimental models have advanced beyond standard ALI monoculture methods to now include the mechanical and physiological parameters of breathing (the cyclic tissue stretch that occurs during inhalation and exhalation) that regulate airflow and pressure, the role of the extracellular matrix (ECM), and multiple cell types (alveolar, endothelial or smooth muscle cells etc.). To determine the preclinical success of an inhaled therapy, therapeutic dosage, efficacy and toxicity must be evaluated in a model(s) that aptly replicate the physiological parameters that influence *in vivo* drug transport mechanisms. Importantly, drug deposition in a specific lung region must first be inhaled via the mouth (oral pharyngeal) and then delivered to the airways (mouth-to-airway transit) and therefore is an important characteristic to replicate and include in preclinical screening models for inhaled therapies. Thus, the use of sophisticated 3-dimensional (3D) experimental models [organoids (Figure 1) and chip-based platforms (Figure 2)] and devices that model mouth-to-airway transit [Andersen Cascade impactor (ACI) (Figure 3), next-generation impactor (NGI) (Figure 4), and twin stage impinger (TSI) (Figure 5)] have advanced the preclinical screening capabilities of novel inhaled therapies.

### Respiratory Organoids Recreate the 3-Dimensional Microenvironment of Airways to Evaluate Drug Efficacy and Toxicity

Respiratory organoids are 3D tissue-engineered culture systems capable of mimicking essential structural aspects of airways to screen drug pharmaceutical safety and efficacy (Jung et al., 2019; Liu et al., 2020). Unlike ALI monoculture models, respiratory organoids physiologically represent the 3D respiratory airway lumen microenvironment by promoting the growth and differentiation of multiple cell types to mimic the diverse structural branching present in airways (Barkauskas et al., 2017; van der Vaart and Clevers, 2020). Respiratory organoids are generated from primary lung and/or pluripotent stem cells that self-aggregate to form spheroids and then embedded within a complex mixture of extracellular matrix (ECM) proteins (collagens, laminin, and fibronectin) (Barkauskas et al., 2017; Chen et al., 2017; Leeman et al., 2019). Exposure to the ECM proteins provides the architectural and physiological support for sustained cell growth, differentiation, cell-cell and cell-matrix signaling pathways that encourages the correct spatially organization of heterogenous cell populations (epithelial, alveolar, mesenchymal cells) to generate luminal growth within the organoid (Figure 1A; Jacob et al., 2017; Tan et al., 2017; Miller et al., 2019). Therapeutic compounds are microinjected directly into the lumen of the organoid (Hill et al., 2017), organoids are integrated within microfluidic devices (via drug diffusion in solution) (Jung et al., 2019) or implanted within *in vivo* models (patient-derived xenografts) (Figure 1B; Tan et al., 2017; Berkers et al., 2019; Kim et al., 2019; Takahashi et al., 2019) to provide a physiologically relevant *in vitro* drug screening platform.

Respiratory organoids have been used to model the specific characteristics and physiological properties numerous respiratory diseases including to cystic fibrosis (Berkers et al., 2019; Dekkers et al., 2013; Liu et al., 2020), fibrosis (Strikoudis et al., 2019), viral and bacterial infections (Chen et al., 2017; Hill et al., 2017; Paolicelli et al., 2019), and lung cancer (Jung et al., 2019; Kim et al., 2019; Takahashi et al., 2019). The delivery of novel preclinical drug compounds to organoid models of respiratory diseases to determine therapeutic efficacy and cytotoxicity has been achieved in a variety of experimentally diverse setups. Hill et al. (2017) delivered drugs directly to the lumen by microinjection (using thin wall glass capillaries) to determine patient-specific drug transport, permeation across an epithelial barrier, efficacy, and toxicity, while preventing luminal contamination of the external growth media. Organoid integration within microfluidic devices has achieved drug delivery under flow conditions, mimicking the pulmonary system (Jung et al., 2019). By implementing flow conditions, a stable supply of nutrients and oxygen is delivered to the organoid but also allowed drug-containing medium to be delivered to the organoid thus, mimicking the diffusion uptake process of systemically delivered drugs. The study by Jung et al. (2019) developed patient-derived lung cancer organoids loaded within a microfluidic chip device to allow clinically relevant chemotherapeutic sensitivity under physiologically relevant flow conditions and determining safe therapeutic concentrations at the preclinical level.

Respiratory organoid models have also been developed to advance personalized medicine, especially for those diseases with high phenotypic and genetic variability including cystic fibrosis (Dekkers et al., 2013; Berkers et al., 2019) and lung cancer (Kim et al., 2019; Kondo and Inoue, 2019). Kim et al. (2019) derived lung cancer organoids from 36 patient tumor tissues of five different histological subtypes to determine individualized patient sensitivities, and in some cases previously unknown resistance to specific chemotherapeutics was found. The direct implantation of organoids *in vivo*, typically mice (known as PDX models), recapitulate the structural hallmarks of cancer tissue and maintain both genetic and histological characteristics of cancer. While drug delivery directly to the luminal space of the organoid is currently unavailable in these PDX models, drugs can be administered via intraperitoneal injection and the organoid then harvested for further pharmacodynamic and bio-pharmacokinetic analyses. Cystic fibrosis organoids been derived from patients with diverse genetic mutations and treatment regimens have been developed to better predict therapeutic outcomes of individual patients (Dekkers et al., 2013; Berkers et al., 2019). Drugs administrated to the culture media of patient-derived cystic fibrosis organoids showed that this was a powerful predictor of therapeutic outcomes and patient responses to single and combination drug treatment regimens for a personalized medicine approach (Dekkers et al., 2013). Thus, patient-derived organoids offer a unique *in vitro* approach to developing personalized, targeted inhalable therapies as they take into account patient-specific phenotypic differences (i.e., mucous production in cystic fibrosis patients or genetic mutations in lung cancer patients) known to impact drug uptake.

Respiratory organoid models serve as a tool to study disease-relevant and physiologically relevant cell-cell interactions when testing the therapeutic efficacy and toxicity of novel drug compounds. However, respiratory organoids lack not only the breathing mechanics that regulate airflow and pressure but also the anatomical structure that gives rise to the mouth-to-airway transit, both vital in determining the aerodynamic performance, pharmacodynamics and deposition of an inhaled therapy. Thus, while organoids are a novel and advantageous *in vitro* tool, they may not be the ideal research tool for screening therapeutic efficacy and toxicity of inhaled compounds as breathing mechanics and lung anatomy are vital to properly assessing the therapeutic efficacy of inhaled compounds.

### Chip-Based Devices Mimic the Physiological and Mechanical Properties of a Lung to Better Predict Therapeutic Outcome of Inhaled Therapies

Organ-on-a-chip-based devices have been engineered to appropriately mimic the physiological and mechanical parameters that regulate and influence, organ homeostasis and function. In terms of the respiratory system, lung-on-chip (LOC) devices simulate the diverse physiological and mechanical parameters of an *in vivo* respiratory environment by replicating the *in vivo* breathing mechanics (the cyclic stretch of inhalation and exhalation), airflow and air pressure dynamics, cellular heterogeneity and vascular flow rates. At the cellular level, it is known that exposure to mechanical stressors, including stretch and/or changes to airflow and fluid shear stress induce proliferation (Gudipaty et al., 2017), differentiation (Edwards, 2001) and cell function (Ke et al., 2019), highlighting the importance of integrating mechanical stress when mimicking an *in vivo* environment. Thus, LOC devices have permitted *in vitro* investigations to determine the behavior of inhaled therapies (aerodynamic performance, bio-pharmacokinetics, therapeutic efficacy and toxicity) and how physiological and mechanical *in vivo* parameters impact drug uptake.

LOC devices are 3D, micro-fabricated, microfluidic devices that feature two separate chambers [an apical (top) and a basal (bottom) chamber] separated by a thin porous membrane that supports the growth, maturation and/or differentiation of distinct cell types on each side of the membrane (Figure 2). Importantly, each chamber can be subjected to independent dynamic flow conditions (i.e., airflow and liquid flow conditions), to better replicate the *in vivo* interplay between cell-cell, cell-matrix and cell-mechanical forces *in vitro* (Huh et al., 2010; Benam et al., 2016). The first LOC device was published by Huh et al. (2010) and reproduced key physiological and mechanical parameters of the human alveolar-capillary interface with alveolar cells seeded on the apical surface of the membrane and exposed to air with variable flow and pressure rates to simulate breathing. Pulmonary endothelial cells were applied to the basal surface of the membrane and subjected to the liquid-filled chamber under dynamic perfusion to mimic blood flow (Huh et al., 2010). The LOC device is uniquely fitted with a vacuum chamber at either side of the apical and basolateral chambers (Figure 2) to simulate the mechanical stretch that occurs during breathing. Taken together, LOC devices provide a more sophisticated ALI model that allows researchers to mimic the *in vivo* pulmonary interactions of the alveolar air space and blood vessels while integrating mechanical stimuli to better model drug deposition and therapeutic studies.

LOC devices model the biological, physiological and pathophysiological characteristics of the human respiratory system and therefore, have been used to assess cellular response(s) following exposure to therapeutic stimuli under dynamic flow conditions. COPD LOC devices have been engineered to measure cellular inflammatory responses to novel anti-inflammatory compounds delivered via the vascular channel (basal chamber), replicating systemic drug delivery (Benam et al., 2016). LOC devices have also been used to model cellular responses to inhaled nanoparticles (delivered apically, in solution) revealing that alveoli cells under cyclic mechanical strain show enhanced cytotoxicity and inflammation in response to nanoparticles in comparison to conventional 2D culture systems that do not possess mechanical force regimens (Huh et al., 2010). Interestingly Huh et al. (2010) also identified that cyclic strain enhanced epithelial and endothelial uptake and therapeutic efficacy of nanoparticles, confirming the importance of integrating mechanical parameters in *in vitro* models.

Chip-based devices have diversified from the single organ on a chip (i.e., LOC) to now include body-on-a-chip (BOC) [sometimes referred to as multi-organ-on-a-chip (MOC)] technologies that can be used to predict the complex organ cross-talk that occurs during drug delivery, metabolism and toxicity (both local and systemic). Concerning the respiratory system, exposure to inhaled therapies and/or toxicants enhances the complexity and physiological relevance of multiple tissue responses, including liver and kidney. The co-culture of 3D respiratory organoids with liver spheroid cultures integrated within a single chip device saw the arrival of the lung/liver-on-a-chip BOC device (Bovard et al., 2018). By incorporating the liver model, Bovard et al. (2018) were able to test both the toxicity of inhaled compounds and metabolites and further monitor the toxicity profile of the administered compounds in both organs *in vitro* (Bovard et al., 2018). Similarly, Miller et al. (2020) engineered a multi-organ breast cancer BOC device to compare the efficacy and toxicity of inhaled and/or intravenously delivered anticancer drug, curcumin. For physiological relevance, the breast cancer BOC device integrated both the liver (complete with recirculating flow), and the lung (including ALI and breathing mechanics to replicate gas exchange, and contraction and expansion) (Miller et al., 2020). Interestingly, curcumin was only found to significantly induce lung toxicity when the device was under static conditions but when breathing mechanics were added to the device, lung cell viability was not affected. Taken together, both the Bovard et al. (2018) and Miller et al. (2020) studies have highlighted the importance of appropriately mimicking the *in vivo* physiological environment to determine therapeutic efficacy and cytotoxicity but also determine the involvement and cross-talk of multiple organs.

LOCs and BOCs offer tremendous potential to be utilized as a dynamic inhaled drug screening platform, with future opportunities to integrate clinically relevant aerosolized inhalation therapy exposure systems as the current mode of drug treatment relies on the direct delivery of solubilized drug into the apical chamber. While LOCs and BOCs mimic the mechanical strain of breathing and the respiratory air-liquid microenvironment, these platforms are not optimized to study “real” aerosol characteristics and interactions during mouth-to-airway transit of drug particles. Importantly, the anatomical and physiological elements of the human respiratory system (including the upper airways: mouth, pharynx and larynx and the lower airways: bronchial branching, mucosal lining, and humidity) need be integrated within *in vitro* models to allow for greater clinical relevance when assessing inhalation therapies.

### *In vitro* Mouth-to-Airway Transit Delivery Models

The anatomical and physiological elements of the human respiratory system (including bronchial branching, mucosal lining, and humidity) need be integrated within *in vitro* models to allow for greater clinical relevance. Both the upper and lower airways contribute to the challenges that govern targeted delivery and disposition of inhaled particles of therapeutic concentration at a specific region of the lung (bronchial epithelium, alveolar space, smooth muscle) can be problematic as significant concentrations of the drug is lost in the upper airways, i.e., during mouth-to-airway transit (Ong et al., 2015). Furthermore, mouth-to-airway transit of inhaled compounds dictates the aerosol deposition at specific anatomical lung regions, an important characteristic to model *in vitro* for *in vivo* relevance. To model aerosol behavior in a physiological and anatomical realistic environment, various impactor technologies (Anderson Cascade impactor, next-generation impactor, twin stage impinger) have been developed and then modified to couple with cell culture models, allowing greater *in vivo* relevance of drug deposition and therapeutic efficacy *in vitro*. While previous reviews have extensively described the use of impactor technologies as inhalation therapy models for drug delivery (Marple et al., 2003a, b, 2004; Haghi et al., 2014a), this section of our review will focus on the advances in these technologies. Conventional *in vitro* impactor studies have utilized ALI culture models to measure the permeability of a drug solution once pipetted onto the cell layer but overlook the important physiochemical characteristics of aerosolized particles (size, surface chemistry and morphology) that affect *in vivo* drug deposition, therapeutic efficacy and therefore clinical relevance. Thus, attempts have been made to modify conventional impactors to study deposition and permeability of aerosolized drugs on respiratory cell culture models.

#### Cascade Impactors

Cascade impactors are multi-staged *in vitro* tools used to characterize aerosol performance at different regions of the lung. Both the Anderson Cascaded Impactor (ACI; Figure 3) and the Next Generation Impactor (NGI; Figure 4) designed to measure the size distribution and concentration of an aerosolized sample under flow conditions. Each stage of a cascade impactor corresponds to a specific region of the lung and therefore the size of the particle dictates the region that the particle can be deposited. For example, Stage 0 corresponds to the mouth and typically allows only those particles with a diameter of less than 10 μm to pass through (cascade) to the following stage. Stage 7 represents the alveolar space and will only allow particles with a diameter of 0.4 μm to enter the stage. Importantly, drug concentration can also be determined at each stage thus, predicting the concentration of drug deposited at specific lung regions.

The ACI is a vertical cascade apparatus and is the primary method for characterizing aerosolized particle deposition of inhaled compounds (Figure 3). Haghi et al. (2014b) developed a modified ACI (mACI) that incorporated ALI models of Calu-3 cells inserted within the mACI at stages 4–7 that represented the deep lung region (the base of the trachea to the alveolar space). By integrating the ALI culture models within the ACI, deposition and subsequent permeability of inhalable Ventolin (salbutamol sulfate) and Qvar (beclomethasone dipropionate) formulations were conducted with drug formulations delivered via an inhaler device [known as a pressurized metered dose inhaler (pMDI)] to provide for clinically relevant administration of inhaled therapies (Haghi et al., 2014b). The mACI model presented in the Haghi et al. (2014b) study demonstrated reproducible and similar patterns of particle deposition when compared to an unmodified ACI, confirming the mACI model as a reliable *in vitro* tool to evaluate micro-particle deposition, permeability and therapeutic efficacy on respiratory epithelia using a delivery system that modeled mouth-to-airway drug transit.

The NGI is a horizontal cascade apparatus used to predict *in vivo* drug behavior that has also been modified to include an ALI cell culture model (mNGIs) able to determine aerosol deposition and transport across cell epithelia (Shur and Price, 2016; van Rensburg et al., 2018; Kumar et al., 2020). mNGIs have been engineered to deliver aerosolized particles under controlled vacuum flow to ALI cultured Calu-3 cells at stages 2, 3, and 4 (van Rensburg et al., 2018) and stages 3, 5, and 7 (Kumar et al., 2020), highlighting the versatility and adaptability of an mNGI depending on a researchers needs. The mNGI has been utilized to examine the uptake of a pMDI administrated aerosolized glucocorticoid (anti-inflammatory) of various particle sizes to determine how particle size influenced not only aerodynamic performance, but also anti-inflammatory efficacy *in vitro* (Kumar et al., 2020). Thus, modified cascade impactors are important and valid *in vitro* drug screening tools, as the integration of cell-based platforms (ALI cultured cells) permit investigations that evaluate *in vivo* deposition of aerosol particles, cellular response and therapeutic efficacy of novel inhaled compounds.

#### Twin Stage Impingers

The modern-day glass twin stage impinger (TSI) apparatus allows aerosolized compounds to be delivered at a simulated oropharynx (mid pharynx) and particles are then separated by size throughout the two stages of the device (Figure 5). The first stage of the TSI replicates the upper airways, while the second stage resembles the lower airways. It is important to note that the TSI does not fully simulate the mouth-to-airway transit as the drug is delivered at the mid pharynx rather than a simulated oral pharyngeal orifice however, the aerosolized fraction that is delivered and collected in the first stage of the TSI has been shown to positively correlate with drug amounts collected in the mouth and throat (Hallworth and Westmoreland, 1987). Regardless, the TSI remains an important and widely used *in vitro* tool to determine the aerodynamic performance of an inhaled therapeutic as particle trajectories can be determined to identify the site-specific deposition of an aerosolized drug (Mendes et al., 2009). Similar to the mNGI and mACI, a modified version of the TSI (mTSI) has integrated an ALI cell culture model at the base of stage 2 (Figure 5) enabling aerosolized drugs (delivered via a pMDI) to be deposited directly at the respiratory epithelia to better mimic *in vivo* biopharmaceutical processes (Figure 5; Grainger et al., 2012; Haghi et al., 2012; Ong et al., 2014). The integration of a microfluidic device as a nebulization platform for pulmonary drug delivery alongside a TSI has been implemented in a study by Qi et al. (2009), demonstrating the use of microfluidics as an efficient means to generate appropriate aerosolized drug particles. A TSI was utilized to confirm the mean aerosol diameter produced by the device, demonstrating the high efficiency of drug particle delivery and the overall viability of the microfluidic platform for inhalation therapy (Qi et al., 2009). It would be of great interest to further enhance and modify TSI models by incorporating alternative *in vitro* cell culture systems (organoids, LOC/BOC devices etc.) to better mimic the respiratory microenvironment for evaluating inhaled drug particle transport, absorption and bio-pharmacokinetics.

The use of *in vitro* cellular models alone, or in combination with microfluidic devices or mouth-to-airway transit models, has increased in our knowledge of cellular behavior following exposure to inhaled compounds. However, cell-based approaches alone or integrated, are unable to holistically mimic the structural and mechanical diversity of a human lung that determine the biological and physiological characteristics of respiration. While the obvious solution is to go directly to *in vivo* rodent and pig models, the translational data acquired from animal respiratory disease models are limited in providing mechanistic insight into human pathologies (Ware, 2008), highlighting the demand for alternative research platforms that better recapitulate the dynamic architecture and complex cellularity of a human lung. Thus, inhaled drug delivery investigations conducted on whole lung tissue (*ex vivo*) models permit a thorough understanding drug behavior *in vivo*, as *ex vivo* lung tissue samples retain *in vivo* airway structure, architecture, cellular heterogeneity and importantly, the microenvironment.

## Advances in *ex vivo* Research Models Offer Translational Preclinical, Therapeutic Relevance When Screening Novel Inhaled Therapies

*Ex vivo* lung models provide opportunities to study the performance and therapeutic efficacy of inhaled drugs in either healthy or diseased lungs. As *ex vivo* lung models retain the 3-dimensional lung structure and native microenvironment (cell-cell, cell-matrix interactions), *ex vivo* drug studies can specifically investigate the role the *in vivo* lung environment plays in determining drug transport kinetics, cytotoxicity and therapeutic efficacy. This next section of the review will focus on two *ex vivo* models, precision-cut lung slices (PCLS) and isolated perfused lungs (IPL) (summarized in Table 2).

**TABLE 2:** Overview of the advantages and disadvantages of *ex vivo* models used for respiratory inhalation drug screening.

*Ex vivo* model	Applications	Respiratory disease model	Advantages	Disadvantages	References
Precision cut lung slices	Drug toxicity and efficacy studies	Pulmonary hypertension	High reproducibility of the platform. Preservation of the lung architecture allowing for a true representation of lung structural response to experimental stimuli. Retain functional cellular interactions for a multicellular response.	Lack of translation of treatment to clinical inhalation applications and dosing evaluation. Inability to mimic ventilation, mechanical stretch, or perfusion observed in human lung.	Paranjpe et al., 2013; Hansen et al., 2016; Hess et al., 2016; Liu et al., 2019
Isolated perfused lungS	Drug delivery/transport and efficacy studies	Severe asthma	Maintains functioning of lung tissue. Intact lung provides for a multicellular response to drug stimuli. Suitable for the direct administration of inhalation drug therapies using inhalable delivery systems. Ability for controlled dosing of drug stimuli.	Typically derived of rodent or rabbit origin which differs in tracheobronchial anatomical and structural composition when compared to human lungs.	Hochhaus et al., 1997; Beck-Broichsitter et al., 2009; Eriksson et al., 2018

### Precision-Cut Lung Slices Maintain Complex Multi-Dimensional and Multi-Cellular Interactions to Appropriately Determine *in vivo* Therapeutic Efficacy

Precision-cut lung slices are sections harvested from lung tissue immediately following *post mortem*, sliced (100–300 μm) and submerged in culture media to retain functional physiological and cellular interactions while maintaining anatomical and structural diversity of a lung (Placke and Fisher, 1987; Liu et al., 2019). This preservation of lung architecture provides for a clinically relevant *ex vivo* respiratory platform to study therapeutic efficacy and cytotoxicity. Thus, PCLS have emerged as a powerful research tool and offer a more complex mechanistic understanding of drug interactions with the multicellular and multidimensional properties of a lung (Hess et al., 2016; Hansen et al., 2016; Cedilak et al., 2019; Liu et al., 2019). In addition, PCLS provide opportunities to assess preclinical, translational studies that determine therapeutic concentrations and efficacies of drugs delivered to different lung regions and have also allowed for cross-species comparisons—with PCLS reportedly prepared from a variety of species including rodent, sheep and human lung tissue (Danov et al., 2018; Lehmann et al., 2018; Yilmaz et al., 2019).

PCLS have been utilized in studies as pre-validation platforms to screen compound toxicity and drug efficacy (Hess et al., 2016; Danov et al., 2018; Cedilak et al., 2019). Drug delivery to *ex vivo* PCLS is often via submersion in drug-containing medium (Nassimi et al., 2009; Neuhaus et al., 2013, 2014; Paranjpe et al., 2013) or direct liquid infusion (via culture medium) to the airway (Hess et al., 2016) as the ventilated delivery of compounds (to mimic inhaled delivery) to PCLS models is currently unattainable. To work around this limitation of PCLS models, Nassimi et al. (2010) administered aerosolized nanoparticles to live BALB/c mice using a jet-driven aerosol generator in a closed Plexiglas box system, mice were then sacrificed and PCLS were generated (Nassimi et al., 2010). Cytotoxicity of PCLS models, regardless of the route of drug delivery, can be assessed by measuring changes to mitochondrial activity, metabolic activity, cytokine release, or imaging of Live/Dead staining of the whole lung section (Nassimi et al., 2010; Neuhaus et al., 2013, 2014; Paranjpe et al., 2013; Hess et al., 2016). PCLS models have further been developed to mimic the complex structure and function relationship of the pulmonary system with connected heart and lung harvested from mice and then sliced (Paranjpe et al., 2013). Paranjpe et al. (2013) determined the therapeutic efficacy of nanoparticles with loaded with Sildenafil (Viagra) as a novel inhalable treatment of pulmonary hypertension. Exposure of the formulated Sildenafil-loaded SLN solutions to the heart and lung slices showed that the IC_50_ value for lung slices was higher than that of heart slices, suggesting that directly targeting the lungs with higher drug concentrations would achieve maximal therapeutic efficacy in the heart and relieve pulmonary hypertension (Paranjpe et al., 2013).

The use of *ex vivo* PCLS models to determine *in vivo* toxicities and therapeutic efficacies are a clinically relevant, pre-validation research platform. As PLCS models consist of whole lung tissue and maintain the complex multi-dimensional and multi-cellular interactions of respiratory, immune and neuronal cells, PCLS models are better placed to properly mimic a therapeutic response to a delivered drug. While PCLS have obvious limitations, namely the inability to mimic ventilation, mechanical stretch or perfusion, this model has been successfully used to determine the preclinical pharmacotoxicology and therapeutic efficacy of novel compounds within an *in vivo* environment (Liu et al., 2019). However, to study the region-specific deposition of inhaled drugs and subsequent transport analysis, an alternative model(s) is required to obtain greater clinical relevance.

### Isolated Perfused Lungs Are a Unique Platform to Determine Inhaled Drug Aerodynamic Performance and Bio-Pharmacokinetics

Isolated perfused lungs are prepared by encasing the whole lung of a rodent or rabbit within an artificial thoracic chamber at physiologically relevant conditions (37°C, 5% CO_2_) and supplying a perfusion buffer (termed perfusate) to mimic pulmonary circulation and ensure the sustained tissue survival and function. In IPL *ex vivo* models, drug agents can be administered directly into the lungs using inhalable delivery systems such as nebulizers, modified nebulizers (AeroProbe), aerosolizers (DustGun) or syringe insufflator (MicroSprayer, PennCentury) (Tronde et al., 2002; Gerde et al., 2004; Ewing et al., 2008; Beck-Broichsitter et al., 2010; Selg et al., 2013) to replicate trachea-to-airway drug transit. However, when replicating mouth-to-airway drug transit in IPL models, delivery devices are limited due to the differences in the oral pharyngeal anatomy between humans and animals. Furthermore, the device selected for inhaled drug delivery or aerosolization are as equally important as the physiochemical properties of the formulation when determining aerosol performance, deposition and efficacy. Nevertheless, *ex vivo* IPL offers a unique opportunity to test inhaled drug delivery in a platform that is physiologically and biologically similar to a human lung allowing drug aerodynamic and bio-pharmacokinetic parameters to be studied.

IPL models are used to determine the uptake of inhaled agents across the pulmonary epithelium, the subsequent rate of drug absorption and therapeutic efficacy (Hochhaus et al., 1997; Bosquillon et al., 2017; Eriksson et al., 2018). IPL has been used to explore the drug transport profiles of inhaled (ventilated)/nebulized compounds, or nanoparticles, that have been formulated to achieve deposition in the tracheal, bronchial or alveolar lung regions with high drug permeability, absorption and retention (Tronde et al., 2002; Ewing et al., 2006; Beck-Broichsitter et al., 2009; Selg et al., 2013; Eriksson et al., 2020). IPL platforms have also integrated sophisticated imaging techniques (photon correlation spectroscopy, laser Doppler anemometry, atomic force microscopy and fluorescence spectroscopy) to allow real-time analysis of drug stability during nebulization, distribution profiles after nebulization (Beck-Broichsitter et al., 2009), further validating IPL as a useful preclinical model for screening novel inhaled drugs.

IPL models are typical of rodent or rabbit origin and it is important that users are aware of the differences in tracheobronchial anatomical and structural composition between animal and human lungs. Not only do the airway diameters of rodent lungs differ to that of human lungs, but rodent lungs are monopodial with asymmetrical branching, while human lungs are dichotomous with symmetrical branching (Phalen et al., 1978; Yeh et al., 1979). While these differences in airway structure have obvious implications for modeling the delivery and deposition of inhaled compounds (Hofmann et al., 1989), IPL offer a generalized understanding of inhaled drug behavior within branched luminal space. It is important to note that IPL models are unable to replicate the initial drug transit mechanisms specifically, inhalation via the oral pharyngeal orifice thus, IPL offers a physiologically relevant platform of the tracheal, bronchial and alveolar regions to screen novel inhaled therapies.

Research platforms used to screen novel inhaled therapeutic compounds, inherently suggests that the research is focused toward finding a treatment for a specific disease or disease phenotype. Yet interestingly, the vast majority of research conducted that utilize IPL as a research tool are conducted on healthy animal lungs. It has long been established that the structural and mechanical properties of diseased lungs alter not only the biological and physiological environment but also the lung capacity (the volume of air able to be inspired and expired) of individuals (Bates et al., 2007). For example, emphysema patients have a significantly reduced lung capacity as a result of the progressive destruction of alveolar spaces and structural properties of the lungs. Therefore, is it important that the relevant mechanical, structural and physiological characteristics of the disease are appropriately modeled to obtain appropriate aerodynamic and bio-pharmacokinetic information when screening preclinical, inhaled therapies.

## Conclusion

There is currently no single faultless universal model for evaluating the delivery, efficacy and toxicity of inhaled therapies thus, a single preclinical model cannot be advocated. The use of multiple, relevant experimental approaches to screen inhaled therapies is essential for the translation of preclinical inhaled drug candidates to clinical practice. The ideal scientific strategy is influenced by numerous factors including the physicochemical properties of the drug, the disease of interest, and the available laboratory resources. Furthermore, when selecting an appropriate model, researchers must understand the limitations and capabilities of *in vitro* and/or *ex vivo* platforms to be implemented in their study. It is therefore critical to understand both the benefits and drawbacks of each scientific platform to appropriately answer the specific research question and to maximize the relevance of the results obtained. Thus, the implementation of multiple experimental platforms provides for greater relevance and validation of models for clinical relevance.

The predictive power of *in vitro* and *ex vivo* correlations for preclinical inhaled therapies has been extensively reviewed in the literature (Sakagami, 2006, 2020; Nahar et al., 2013; Nickel et al., 2016; Ehrmann et al., 2020) and comparisons of *in vitro* and *ex vivo* data have been used to establish, validate and compare experimental models with known *in vivo* responses (Sakagami, 2006, 2020; Nahar et al., 2013; Nickel et al., 2016; Ehrmann et al., 2020). The most common *in vivo* tool used for comparative inhaled therapy studies to date is the ALI culture model, while the most common complementary *ex vivo* model is the IPL platform, with studies demonstrating results that are comparable to *in vivo* data (Nassimi et al., 2010; Ong et al., 2014; Bosquillon et al., 2017). The *in vivo* predictive capabilities of *in vitro* and *ex vivo* methodologies when performed side-by-side have the potential to enhance novel drug screening platforms and bridge the translation from preclinical testing to the patient population. Innovative drug screening platforms can accelerate and facilitate preclinical therapeutic studies to allow for more informed decisions on potential inhalation drug candidates for future use in clinical trials. Thus, the rapidly evolving and innovative research space of 3D *in vitro* and *ex vivo* platforms to model respiratory diseases and evaluate the therapeutic potential of inhalation drug candidates provides promising avenue for screening preclinical inhaled therapies.

## Author Contributions

AC and PB prepared the manuscript. AC and HO prepared tables and figures. HO, PB, and DT provided critical feedback as all authors contributed equally towards conceptualization of the finalized manuscript.

## Conflict of Interest

The authors declare that the research was conducted in the absence of any commercial or financial relationships that could be construed as a potential conflict of interest.
